# Utilization of Cactus Peel as Biosorbent for the Removal of Reactive Dyes from Textile Dye Effluents

**DOI:** 10.1155/2020/5383842

**Published:** 2020-07-25

**Authors:** Mebrahtu Gebrezgiher, Zebene Kiflie

**Affiliations:** ^1^Department of Chemical Engineering, College of Engineering and Technology, Adigrat University, Adigrat, Ethiopia; ^2^School of Chemical and Bio-Engineering, Addis Ababa Institute of Technology, Addis Ababa University, Addis Ababa, Ethiopia

## Abstract

Textile industries generate large quantities of dye containing wastewater which pose a serious environmental problem. Currently, biosorbents have become desirable for the removal of dyes from textile effluents. In this study, batch experiments were conducted to investigate the biosorption characteristics of cactus peel on the removal of reactive red dye from aqueous solutions. The effects of solution pH, biosorbent dosage, contact time, and initial concentration were studied. The interaction effects of process variables were analysed using response surface methodology. The results showed that removal efficiency increased as initial dye concentration and solution pH decreased and as biosorbent dosage and contact time increased. The highest removal efficiency (99.43%) was achieved at solution pH, initial dye concentration, biosorbent dose, and contact time of 3.0, 40 mg/l, 6 g, and 120 min, respectively. From regression analysis, the Langmuir isotherm was found to better (*R*^2^ = 0.9935) represent the biosorption process as compared with the Freundlich isotherm (*R*^2^ = 0.9722). Similarly, the pseudo-second-order model was seen to represent very well the biosorption kinetics. The results show that cactus peel has good potential for the removal of reactive red dye.

## 1. Background

World arithmetic figures show that more than 70% of the surface of the Earth is covered by water. However, a considerable percentage of this water is not suitable for human consumption and aquatic organisms because of different wastes discharged to it. Water contamination occurs due to the growth of industries, increase in population, urbanization, lack of environmental awareness, and untreated effluents discharged from industries and municipalities. Developing nations like Ethiopia are hurt more by water pollution, where already almost 75% of health sickness is directly or indirectly related with lack of potable water. Dyes have numerous applications in industries such as textile, rubber, paper, plastic, leather, and cosmetic. From these various industries, the textile industry levels first in usage of various types of dyes to paint its products. Textile industry is one of the fastest rising industries and expressively helps to the economic growth of a country including Ethiopia. This industry also has high water consumption and at the same time produces high discharge rate of wastewater with high load of dyeing agents and large amount of suspended organic solids.

The availability of little amounts of dyes in water (less than 1 ppm for some dyes) is highly observable and undesirable due to their good solubility [1]. Usually, about 10–25% of textile dyes are vanished during the process of dyeing, and about 2–20% are directly discharged as aqueous wastes in diverse environmental components [2]. Many of these dyes are toxic and carcinogenic. The availability of untreated dye effluents seriously affects the quality of water. Even if at low concentration, reactive dye can cause high prominence and undesirability. Furthermore, color produced due to dyes in water makes it aesthetically disagreeable [3]. They can have acute or chronic properties on aquatic organisms, which depend on the dye concentration and the exposure time [4]. Therefore, their removal from water is a necessity. However, textile wastewater containing dyes is very problematic to treat for the reason that dyes are resistant to aerobic digestion and are stable to light, heat, and oxidizing agents and can remain in the environment for a long period of time owing to their complex chemical structures [4]. Many methods have been recommended and applied for removing dyes from textile industry effluents, including chemical precipitation, ion exchange, filtration, flocculation, and membrane separation. Moreover, these methods have imperfect application as they often involve high capital and operational costs and may have low efficiency that is linked with the generation of secondary wastes such as sludge which needs further treatment or purification [5]. In this regard, biosorption offers advantages over the other methods because of its humble design with a sludge-free environment and its low investment costs. Biosorption is a mass transfer operation in which a substance is transferred from the liquid phase to the surface of a solid (biomass), and the substance becomes adhered by physical and/or chemical interactions. Due to large surface area, high biosorption capacity, and surface reactivity of the biosorbent, it can be exploited as low-cost alternatives [6]. The uses of the biosorbent have been studied for different applications by many researchers. Naddafi and Saeedi studied the biosorption of copper (II) from aqueous solutions using protonated and original *Cystoseira myrica* biomass in which they found out that biosorption with the protonated biomass was rapid but with reduced uptake capacity and concluded that protonation was an inefficient pretreatment method [7]. Mahvi et al. [8] investigated the fluoride biosorption from aqueous solutions using the *Ziziphus* leaf, and their experimental results showed that the *Ziziphus* leaf can be used as an environmentally friendly, cost-effective, and effective biosorbent for the removal of fluoride from aqueous solutions.

Although the exact amount is not known, cactus peel is one of the highly abundant solid byproducts that is generated in many places in Ethiopia, especially in the northern parts. Cactus peel is obtained from cactus fruit which is edible. The peel is composed of cellulose, hemicellulose, pectin, lignin, and gums [9]. In addition, this material consists of 2.3% nitrogen, 29.4% carbon, and 1.7% hydrogen. So, the aim of the present work is to investigate the biosorption capacity of cactus peel for the removal of reactive dyes from textile wastewater.

## 2. Materials and Methods

### 2.1. Equipment and Apparatus

The different pieces of equipment and apparatus used in this work include the analytical balance (Ohaus, EP 214C, Switzerland), grinder, 250 ml plastic bottles, orbital shaker (GFL 3074 model), sieves (200–250 mm size), pH meter (model: Jenway; 3505 pH meter), magnetic stirrer, UV spectrophotometer (Jenway 6300, England), FTIR (model: PerkinElmer Spectrum 65), XRD, drying oven (model: Memmert, 100–800 kg capacity, Germany), furnace (Nabertherm LHT 02/16, Germany), micropipette (10–25 ml), crucible, volumetric flasks (50–250 ml), pipettes, desiccators, test tubes, glove, and Whatman filter paper.

### 2.2. Chemicals and Reagents

The chemicals that were used during this research work include hydrochloric acid (HCl), sodium hydroxide (NaOH) solution, potassium bromide (KBr), and reactive red dye. Powder reactive dye was obtained from Ayka Addis Textile Industry and was used in the experiments without extra purification. The rest of the chemicals were all of analytical grade and were purchased from different chemical suppliers in the Addis Ababa city. The raw material used as biosorbent was cactus peel.

### 2.3. Preparation of Cactus Peel

Fresh cactus peel from cactus fruit was collected from agricultural farm lands around Adigrat, Northern Ethiopia. The peels were washed with tap water and further cleaned with distilled water to remove impurities. Afterwards, the peels were dried in an oven at 90°C for 24 hours and then ground into fine powders using the grinder and sieved through a screen.

### 2.4. Pretreatment of the Biosorbent Material

The ground and sieved cactus peel was soaked in HCl solution and agitated at 200 rpm for 24 hours in order to remove impurities and to improve the biosorbtive capacity of the biosorbent. The biosorbent was then washed with distilled water until the eluate pH reached near to neutral. Then, it was further dried at 105°C for 24 hours and kept in an airtight container until further use in the subsequent batch biosorption studies [10].

### 2.5. Characterization of the Biosorbent

#### 2.5.1. Moisture Content Determination

The moisture content of cactus peel was determined by drying the peel in the oven at 105°C until the dried sample weight remained constant and using the following equation:

(1) $$\begin{array}{c} \text{moisture content}=\;\frac{W_{1}-W_{2}}{W_{1}}\;*\;100, \end{array}$$

where *W*_1_  is the sample weight before drying and  *W*_2_  is the sample weight.

#### 2.5.2. Ash Content Determination

For the ash content determination, a preweighed dry sample, placed on a clean and dry ceramic crucible, was transferred into a muffle furnace and was kept at 500°C for one hour for complete decarbonization. Ash content was then calculated using the following equation:

(2) $$\begin{array}{c} \text{ash content}\left(\%\right)=\frac{W_{\text{f}}}{W_{\text{i}}}*\;100, \end{array}$$

where  *W*_f_  is the mass of the ash and *W*_i_  is the mass of the cactus peel powder placed in the furnace.

#### 2.5.3. Volatile Matter Content Determination

The volatile matter was determined by placing a preweighed dry sample in a preignited muffle furnace at a temperature of 600°C for 15 minutes and using equation (3). The sample was cooled inside a desiccator for 10 minutes before measurement.

(3) $$\begin{array}{c} \text{VM}=\frac{M_{\text{r}}*100}{{\text{M}}_{\text{i}}}, \end{array}$$

where VM is the volatile matter, *M*_r _ is the residual mass after volatilization, and *M*_i_ is the mass of the sample placed in the furnace.

#### 2.5.4. Fixed Carbon Content Determination

The fixed carbon content (FC) was calculated according to the American Society for Testing Materials (ASTM) [11] using the following equation:

(4) $$\begin{array}{c} \text{FC}=100-\left(\text{moisture content}+\text{volatile matter}+\text{ash content}\right). \end{array}$$

#### 2.5.5. Fourier-Transform Infrared Spectroscopy (FTIR) Analysis

FTIR characterization was performed in order to identify the functional groups existing on the biosorbent that might be involved in the reactive dye uptake process. FTIR analysis was conducted on the cactus peel powder before and after biosorption using the PerkinElmer spectrum 65 model FTIR spectrometer in the wavenumber range of 4000 cm^−1^ to 400 cm^−1^.

#### 2.5.6. X-Ray Diffraction (XRD) Analysis

XRD spectra of powdered cactus peel were characterized by using D8 Advance XRD of the Brucker powder diffractometer with Cu-K*α* (*λ* = 1.540593–1.544414 Å, 40 kV, and 15 mA) radiation having a continuous scanning mode with a speed of 10 deg/min in the 2*θ* range of 3° to 90° with the scan step of 0.020. The analysis was performed in College of Natural Sciences, Department of Chemistry, Addis Ababa University.

### 2.6. Stock Solution Preparation

A stock solution of dye (1000 ppm of reactive dye solution) was prepared by dissolving 1 g of powder dye in 1000 ml distilled water. Different concentrations (40 ppm, 60 ppm, and 80 ppm) were prepared by further dilution of the stock solution with distilled water.

### 2.7. Experimental Design

The experimental runs were conducted randomly, and suitable analysis technique has been ensured by the help of Design-Expert® version 7 software. In addition, the central composite design (CCD) was used for fitting a quadratic surface, which usually works well for optimization and also reduces the number of experiments to be conducted [2]. Solution pH, initial dye concentration, biosorbent dose, and contact time were the four independent process factors investigated, while the response (output) variable was the amount of reactive dye removed from the aqueous solutions by the cactus peel powder, hereafter referred to as removal efficiency. Thus, with CCD, four independent variables (*n* = 4) and two central points (nc = 2), each with two different levels, were used. Hence, the total number of experiments (*N*_t_) was calculated from the following equation:

(5) $$\begin{array}{c} N_{t}=2^{n}+2n+\text{nc}. \end{array}$$

## 3. Results and Discussion

### 3.1. Characterization of Cactus Peel

#### 3.1.1. Proximate Analysis

The results of the proximate analysis of powdered cactus peel are summarized in Table 1. From the results, it can be seen that cactus peel has high fixed carbon and a small percentage of ash content. This indicates that cactus peel is suitable for preparing the porous structure, a necessary condition to have increased surface area.

#### 3.1.2. FTIR Analysis

**Figure 1:**
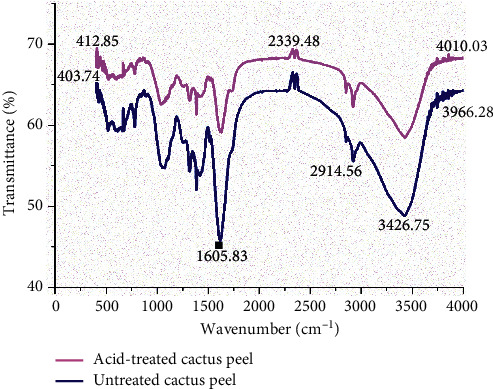
FTIR analysis of acid-treated and untreated cactus peel.

The FTIR spectra of the raw and the acid-treated cactus peel are shown in Figure 1. The spectra in the range 3420–3440 cm^−1^ indicate the existence of O-H stretching vibrations of cellulose, pectin, and lignin and -NH groups on the biosorbent surface. The spectra peaks around 1382 cm^−1^ and 1050 cm^−1^ show the presence of the -C-H bending vibration of alkane and C-O stretching vibration, respectively [12]. The peak around 2921 cm^−1^ might be assigned to -CH stretching vibration functional groups. After HCl treatment, more functional groups like -NH, -OH, C-O, C-H, -CH, and -COOH have become evident.

**Figure 2:**
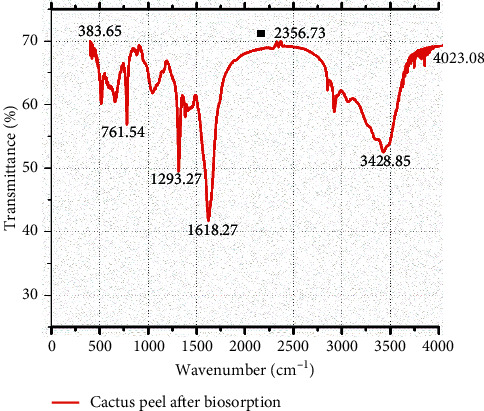
FTIR analysis of cactus peel after biosorption.

Figure 2 displays the FTIR analysis of cactus peel after biosorption. The stretching vibration absorption band at 1616.35 cm^−1^ is assigned to carboxylic groups while that at 3415.38 cm^−1^ shows the presence of O-H. As it can be seen in the same figure, the broad peak representing hydroxyl and amine groups has shifted from 3424.02 to 3415.38 cm^−1^ after the biosorption process indicating the attachment of reactive dye molecules on the available functional groups of the cactus peel biosorbent surface.

#### 3.1.3. XRD Analysis

**Figure 3:**
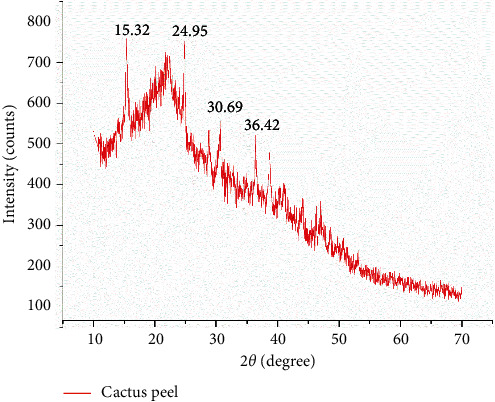
XRD analysis of cactus peel.

Figure 3 shows the characteristic peaks on the powder XRD pattern of the cactus peel biosorbent. The pattern shows a largely amorphous structure. Most of the diffraction peaks for the cactus peel biosorbent are located around the scattering angle (2*θ*) of 15–35°. In contrast, the sharp peaks at scattering angles (2*θ*) of 15.36, 24.93, 28.75, 30.58, 36.35, and 38.56° are seen to be not very strong indicating that the crystalline portion present is small. From this, it can be concluded that the material is suitable for preparation of a porous material.

### 3.2. Linear and Interaction Effects of Different Factors Based on CCD

The effects of four independent variables, i.e., solution pH (3 ≤ pH ≤ 9), initial dye concentration (40 mg/L ≤ con ≤ 80 mg/L), biosorbent dose (2 g ≤ dose ≤ 6 g), and contact time (40 min ≤ time ≤ 120 min), on removal efficiency were evaluated using CCD. Statistical significance of the main and interaction effects of all the variables was predicted by analysis of variance (ANOVA).  Table 2 gives the experimental design data and the corresponding experimentally determined removal efficiencies.

The model equation for the dye removal is given in the following equation:

(6) $$\begin{array}{c} \text{dye removal efficiency}=72.35-6.52\text{A}-8.99\times \text{B}+7.41\text{C}+2.37\text{D}-2.15\text{AB}+1.79\text{AC}+2.49\text{BC}-1.71\text{BD.} \end{array}$$

This model includes the linear and the interaction terms. As shown in Table 3, all the linear terms have statistical significance (*P* < 0.05). As regards the interaction terms, the effects of solution pH and time as well as those of dose and time were not found to be statistically significant (*P* < 0.05).

**Figure 4:**
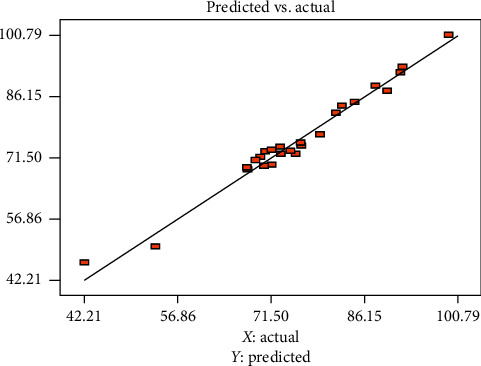
Actual vs. predicted response of the reactive red dye removal efficiency of cactus peel.

Figure 4 also shows the actual vs. predicted responses for the biosorption of reactive red dye with cactus peel. The linear relationship between the predicted and the actual with the coefficient of determination (*R*^2^) of 0.976 shows good agreement between model prediction and actual removal efficiency. Thus, the model can give information on the linear and the interaction effects of the different factors.

### 3.3. Linear Effects of Different Factors on the Removal Efficiency of Cactus Peel

The separate effects of initial dye concentration, biosorbent dosage, and contact time on the removal efficiency of cactus peel were examined.

Initial dye concentration is important since a given mass of the biosorbent material can only adsorb a fixed amount of dye [13]. Experiments carried out at pH of 6, biosorbent dose of 4 g, and contact time of 80 minutes in order to investigate the effect of initial dye concentration on removal efficiency (results not shown here) indicate that the removal efficiency increases with decrease in initial dye concentrations. At lower dye concentrations, the ratio of biosorbent active sites to the amount of solute is higher which favors increase in color removal [14]. On the contrary, at higher initial dye concentrations, more active sorption sites would be occupied by the dye ions and become saturated.

The effect of changes in biosorbent dosage on removal efficiency was also investigated at solution pH of 6, initial dye concentration of 60 mg/L, and contact time of 80 min. The results (not shown here) indicate that, with increase in biosorbent dosage, the removal efficiency increases which may be due to the availability of increased biosorption surface area with increased biosorbent dosage [15]. Also, the effect of contact time on biosorption of reactive dye using cactus peel was studied by keeping the other variables (solution pH of 6, initial dye concentration of 60 g/L, and biosorbent dosage of 4 g) constant. At these conditions, the removal of dye was found to increase with increase in contact time to some extent. The reason might be that the solutes get more time to access the inner active sites as the contact time increases. However, further increase in contact time did not increase the removal efficiency, probably, due to deposition of dye molecules on the available active sites of the biosorbent material [16]. Similar observations can also be made based on the statistical data shown in Table 3. Here, we can see that all the linear terms (solution pH, initial dye concentration, biosorbent dosage, and contact time) have statistically significant effect (*P* < 0.0001) on the removal efficiency. Moreover, it is possible to see from the model equation (equation (6)) that the removal efficiency decreases with increase in solution pH and initial dye concentration and increases with biosorbent dosage and contact time. The model equation also indicates that the effect of solution pH is by far the most important. This may be because at lower pH, the positive charges which dominate the surface of the biosorbent promote the biosorption of the reactive red dye. Similar findings are reported by different authors [17, 18].

### 3.4. Effects of Interaction Parameters on Percentage Dye Removal

#### 3.4.1. Interaction Effects of Solution pH and Initial Dye Concentration

**Figure 5:**
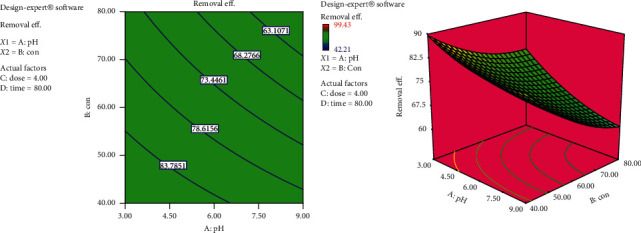
Interaction effects of solution pH and initial dye concentration on removal efficiency (dose = 4 and time = 80 min).

The interactive effects of solution pH and initial dye concentration on the removal efficiency are given in Figure 5, where the contour and the surface plots are shown. The figure shows that, as both solution pH and initial dye concentration increase, the removal efficiency decreases and vice versa. The same effect is also reflected in model equation (6). From this, we can conclude that solution pH and dye concentration have strong effect on the removal efficiency. Moreover, a close scrutiny of the figure reveals that the decrease in removal efficiency with increase in dye concentration is more pronounced at lower pH than at higher pH values. In fact, the highest removal efficiency is observed at the lowest values of pH and initial concentration as shown in Figure 5.

#### 3.4.2. Interaction Effects of Initial Dye Concentration and Contact Time

**Figure 6:**
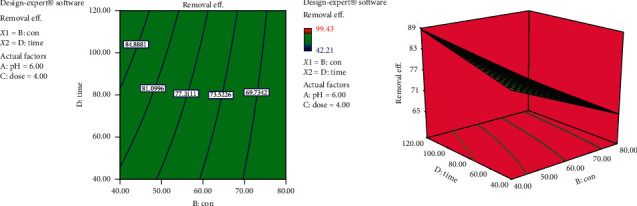
Interaction effects of initial dye concentration and contact time on removal efficiency (pH = 6 and dose = 4 g).

The interaction effect of initial dye concentration and contact time on biosorption of reactive dye by the cactus peel biosorbent is illustrated in Figure 6. From this graph, it was concluded that biosorption of reactive dye increases with the increase in the contact time to a certain extent. Further increase in contact time does not increase the dye uptake process due to deposition of dyes on the available active sites of the biosorbent material. In addition, it can be seen that the removal efficiency decreases with increase in the initial dye concentration which may be due to the fact that, with higher initial dye concentrations, the active sites of the biosorbent get occupied by the dye ions and become saturated.

#### 3.4.3. Interaction Effects of Biosorbent Dose and Contact Time

**Figure 7:**
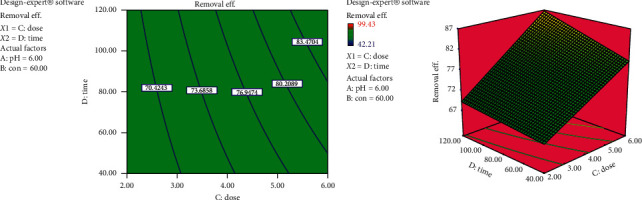
Interaction effects of biosorbent dose and contact time on dye removal efficiency (pH = 6 and con = 60 mg/L).

From Figure 7, it is evident that the removal efficiency increases as both biosorbent dose and contact time increase. In other words, both have a positive effect. The effect of adsorbent dose is obvious since increasing biosorbent dose would make higher number of biosorption sites available. The effect of contact time is as explained earlier. However, it is worth to note that the effect of increasing dosage on the removal efficiency is higher at lower contact times. This may be explained by the fact that, at lower contact time, the driving force is higher and gradually decreases with time as equilibrium is approached.

### 3.5. Dye Biosorption Isotherm Models

Isotherm models designate how the adsorbed molecules dispense themselves between the liquid phase and the solid phase when the biosorption process reaches equilibrium. They can give information on how the biosorption process proceeds and can be used to observe how efficiently a given biosorbent interacts with the adsorbate [19]. The most widely known surface biosorption isotherm models for single-solute systems are the Langmuir and Freundlich models [20]. So, in this study, the adequacy of the models to represent the experimental data is tested using regression analysis.

#### 3.5.1. Langmuir Isotherm Model

The Langmuir model is based on the assumption that the maximum adsorption occurs when a saturated monolayer of solute molecules is present on the adsorbent surface. The model assumes that all the sorption sites have equal affinity for molecules of the adsorbate (solute), and there is no transmigration of the adsorbate in the plane of the surface [21].

The model can be expressed in the following linear form:

(7) $$\begin{array}{c} \frac{C_{\text{e}}}{Q_{\text{e}}}=\frac{1}{Q_{\text{m}}b}+\frac{C_{\text{e}}}{Q_{\text{m}}}, \end{array}$$

where *C*_e_ is the equilibrium concentration (mg/l), *Q*_e_ is the amount adsorbed at equilibrium (mg/g), *Q*_m_ is the Langmuir constant related to adsorption capacity (mg g^−1^), and *b* is the Langmuir constant related to the energy of adsorption (L mg^−1^).

**Figure 8:**
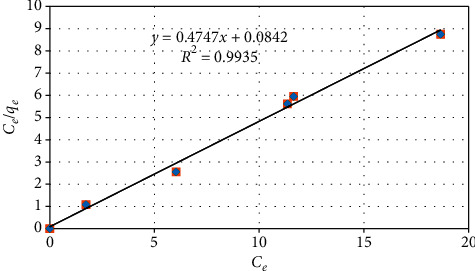
Langmuir isotherm model plot.

Thus, if the experimental data are described by the Langmuir isotherm, then a plot of *C*_e_/*Q*_e_ vs. *C*_e_ should be linear. Hence, the values of *Q*_m_ and *b* will be determined from the slope and the intercept of the plot of Figure 8, respectively. Figure 8 is plotted using the equilibrium and calculated data shown in Table 4.

#### 3.5.2. Freundlich Isotherm Model

The Freundlich isotherm is based on multilayer adsorption on the heterogeneous surface of the biosorbent containing an unequal amount of energies [22].

The linear form of the Freundlich equation is expressed as

(8) $$\begin{array}{c} \log\;q_{\text{e}}=\frac{1}{n}\log\;c_{\text{e}}+\log\;k_{\text{f}}, \end{array}$$

where *q*_e_ is the amount of dye ions biosorbed per unit weight of the biosorbent (mg/g), *c*_e_ is the equilibrium concentration in solution (mg/l), *k*_f_ and 1/*n* are the Freundlich constants, and *k*_f_ and *n* are the indicators of the biosorption capacity and biosorption intensity, respectively.

**Figure 9:**
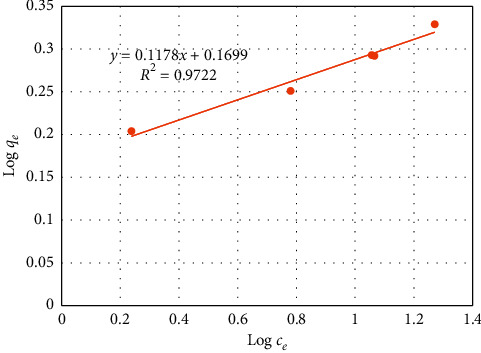
Freundlich isotherm model plot.

The adequacy of the Freundlich model to fit the experimental data was examined from a plot of log (*q*_e_) vs. log (*c*_e_) as shown in Figure 9 using calculated data from Table 4. From Figure 9, the calculated values of *k*_f_ and *n* are 1.4787 and 8.48, respectively. Values of “*n*” lying in the range of 1–10 indicate favorable biosorption. The intensity parameter, 1/*n*, indicates the deviation of the isotherm from linearity. A smaller value of 1/*n*, therefore, shows a better biosorption mechanism and formation of a stronger bond between the solute and the solid biosorbent [23]. In light of this, it can be said that the equilibrium isotherm of the biosorption of reactive red dye on cactus peel is characterized by favorable equilibrium. In addition, the value of 1/*n*, which is 0.118, implies that the bonding between the dye and the peel is strong.

The model constants of these two isotherm models, as obtained from the slopes and intercepts of the plots of Figures 8 and 9 as well as the respective regression coefficients, are given in Table 5. Thus, comparing the regression coefficients of the two plots, one can see that the biosorption data are better fitted by the Langmuir isotherm model (*R*^2^ = 0.9935) than by the Freundlich isotherm model (*R*^2^ = 0.9722). In addition, based on the Langmuir model, the maximum removal capacity (within the investigated experimental range) is estimated at 2.11 mg/g.

### 3.6. Dye Biosorption Kinetics

The sorption mechanism and the rate of the biosorption process are important for assessing the biosorption process. To use cactus peel efficiently as a potential biosorbent, contact time is of fundamental importance. In this regard, the cactus biosorption of reactive red dye kinetics was investigated using the two common kinetic models, i.e., the pseudo-first-order and pseudo-second-order models.

#### 3.6.1. Pseudo-First-Order Kinetic Model

The pseudo-first-order equation is given as

(9) $$\begin{array}{c} \frac{\text{d}q}{\text{d}t}=k_{1}\left(q_{\text{e}}-q_{\text{t}}\right). \end{array}$$

After integration with the boundary conditions *q*_t_ = 0 at *t* = 0 and *q*_t_ = *q*_t_ at *t* = *t*, the model can be expressed as

(10) $$\begin{array}{c} \text{Log }\left(q_{\text{e}}-q_{\text{t}}\right)=\text{Log }q_{\text{e}}-\left(\frac{k_{1}}{2.303}\right)t, \end{array}$$

where *q*_e_ is the biosorption capacity at equilibrium, *q*_t_ is the biosorption capacity at any time *t*, and *k*_1_ is the rate constant of the pseudo-first-order biosorption model (min^−1^).

**Figure 10:**
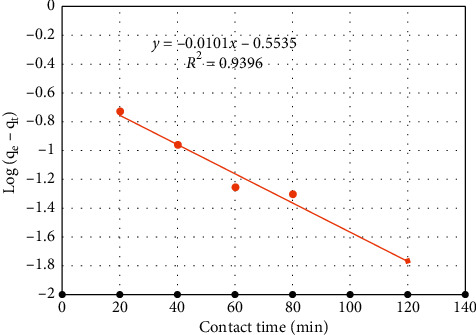
Pseudo-first-order model linear fit for biosorption of reactive dye onto cactus peel.

Thus, a linear plot of log (*q*_e_ − *q*_t_) against *t* can indicate how best the model describes the experimentally observed kinetics. Accordingly, log (*q*_e_ − *q*_t_) vs. *t* is plotted using the corresponding data from Table 6 and shown in Figure 10.

#### 3.6.2. Pseudo-Second-Order Kinetic Model

The pseudo-second-order model is based on the assumption that the biosorption may be second order. The pseudo-second-order biosorption kinetic rate equation is expressed as

(11) $$\begin{array}{c} \frac{\text{d}\left(q_{\text{t}}\right)}{\text{d}t}=k_{2}{\left(q_{\text{e}}-q_{\text{t}}\right)}^{2}. \end{array}$$

Integrating and rearranging the above equation (at *t* = 0, *q*_t_ = 0 and at *t* = *t*, *q*_t_ = *q*_t_), we get the following equation, where a plot of t/qt vs. *t* should lie on a straight line:

(12) $$\begin{array}{c} \frac{t}{q_{\text{t}}}=\frac{1}{\left(k_{2}q_{\text{e}}^{2}\right)}+\frac{t}{q_{\text{e}}}, \end{array}$$

where *q*_e_ is the amount of dye biosorbed at equilibrium, (mg/g), *q*_t_ is the amount of dye biosorbed at any time *t*, and *k*_2_ is the rate constant of sorption.

**Figure 11:**
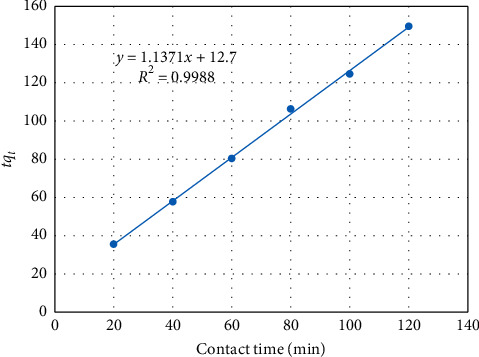
Pseudo-second-order linear fit for biosorption of reactive dye onto cactus peel.

Figure 11 demonstrates the plot of *t*/*q*_t_ vs. contact time using the corresponding data given in Table 6. The predicted equilibrium biosorption capacity (*q*_e_) and the pseudo-second-order rate constant (*k*_2_) were calculated from the slope and intercept of the plot of Figure 11, respectively. The rate parameters for the two models, the correlation coefficients (*R*^2^) and the predicted *q*_e_ values, are presented in Table 7.

Comparing the two kinetic models based on the correlation coefficients, we can see that the experimental data poorly fitted with the first-order kinetic model. In addition, the pseudo-first-order kinetic model predicted a very low value of the equilibrium sorption capacity (*q*_e_ = 0.2796 mg/L) implying that the sorption kinetics cannot be properly described by the pseudo-first-order model. On the contrary, the experimental data are found to fit very well with the pseudo-second-order model. Furthermore, the second-order model predicted an equilibrium sorption capacity (*q*_e_) of 0.8789 mg/l which is in good agreement with the experimentally found *q*_e_ (*q*_exp_ = 0.803). Thus, it can be said that the biosorption kinetics of the reactive red dye on cactus peel can be adequately represented by the pseudo-second-order kinetic model.

## 4. Conclusions

In this research work, the biosorption of reactive dye from aqueous solution using powder cactus peel as the biosorbent was investigated. The separate and interaction effects of solution pH, initial dye concentration, contact time, and biosorbent dose were studied. The results show that all linear terms have significant effect on the removal efficiency. Some interaction effects were also statistically significant.

The Langmuir isotherm model represents the experimentally obtained equilibrium data better. Similarly, the pseudo-second-order kinetic model was seen to describe the observed kinetic behavior of the biosorption process. The maximum removal obtained was 99.43% which was achieved at pH of 3.0, biosorbent dose of 6.0 g, initial dye concentration of 40 mg/L, and contact time of 120 minutes. These results indicate that acid-treated powder cactus peel could be used for the removal of reactive red dye from aqueous solution. Therefore, with the abundance of cactus trees, and hence cactus peel, it is worth to investigate the effect of other solutes and the performance of this biosorbent using column studies.

## Figures and Tables

**Table 1:** Proximate analysis of cactus peel.

Contents	Value (%)
Moisture content	2.63
Ash content	1.56
Volatile matter	7.22
Fixed carbon	88.59

**Table 2:** Experimental design data for batch biosorption of reactive red dye with cactus peel.

Run	Factors				Response
	A: pH	B: conc.(mg/L)	C: dose (g)	D: time (min)	(%) removal
1	3.00	40.00	2.00	40.00	82.68
2	9.00	40.00	2.00	40.00	72.99
3	3.00	80.00	2.00	40.00	67.84
4	9.00	80.00	2.00	40.00	53.37
5	3.00	40.00	6.00	40.00	89.76
6	9.00	40.00	6.00	40.00	84.67
7	3.00	80.00	6.00	40.00	81.73
8	9.00	80.00	6.00	40.00	69.06
9	3.00	40.00	2.00	120.00	91.86
10	9.00	40.00	2.00	120.00	79.25
11	3.00	80.00	2.00	120.00	71.65
12	9.00	80.00	2.00	120.00	42.21
13	3.00	40.00	6.00	120.00	99.43
14	9.00	40.00	6.00	120.00	92.13
15	3.00	80.00	6.00	120.00	87.94
16	9.00	80.00	6.00	120.00	75.38
17	5.70	60.00	4.00	80.00	76.29
18	6.30	60.00	4.00	80.00	70.62
19	6.00	58.00	4.00	80.00	76.27
20	6.00	62.00	4.00	80.00	71.68
21	6.00	60.00	3.80	80.00	69.82
22	6.00	60.00	4.20	80.00	74.56
23	6.00	60.00	4.00	76.00	67.74
24	6.00	60.00	4.00	84.00	70.49
25	6.00	60.00	4.00	80.00	73.07
26	6.00	60.00	4.00	80.00	73.09

**Table 3:** ANOVA table for the response surface quadratic model of biosorption of reactive red dye.

	*P* value
A	<0.0001
B	<0.0001
C	<0.0001
D	0.0053
AB	0.0093
AC	0.0243
AD	0.0954
BC	0.0039
BD	0.0295
CD	0.0748
Lack of fit	0.0038
R-squared	0.976

**Table 4:** Equilibrium and calculated data for Langmuir and Freundlich isotherm model plots (solution initial pH = 4.0, biosorbent dose = 6.0 g, and contact time = 120 min).

*C*_e_ (mg/L)	*q*_e_ (mg/g)	Langmuir isotherm model	Freundlich isotherm model	
		*C*_e_/*q*_e_	Log (*C*_e_)	Log (*q*_e_)
11.3465	1.963	5.62	1.055	0.293
6.0256	2.245	2.56	0.78	0.251
18.6636	2.133	8.75	1.271	0.329
11.6412	1.958	5.95	1.066	0.292
1.7298	1.601	1.08	0.238	0.204

**Table 5:** Langmuir and Freundlich isotherm model parameters.

Langmuir isotherm			Freundlich isotherm		
*Q*_m_ (mg/g)	*b* (L/mg)	*R* ^2^	*k*_f_	*n*	*R* ^2^
2.11	5.637	0.9935	1.4787	8.48	0.9722

**Table 6:** Kinetic and calculated data for pseudo-first-order and pseudo-second-order kinetic model plots (solution initial pH = 4.0 and initial dye concentration = 40 mg/L).

Time (min)	Final dye concentration (mg/l)	*q*_t_ (mg/g)	Log (*q*_e_ − *q*_t_)	*t*/*q*_t_ (min/(mg/g))
20	15.383	0.615	−0.725	35.52
40	12.262	0.693	−0.958	57.72
60	10.114	0.747	−1.252	80.32
80	9.845	0.753	−1.301	106.24
100	7.899	0.803	—	124.53
120	7.899	0.803	—	149.44

**Table 7:** Model fit parameters related to the kinetic models.

Pseudo-first-order model			Pseudo-second-order model			q_exp_ (mg/g)
*q*_e_ (mg/g)	*k*_1_ (min^−1^)	*R* ^2^	*q*_e_ (mg/g)	*k*_2_ (g·mg^−1^min^−1^)	*R* ^2^	
0.2796	0.2336	0.9396	0.8789	0.1019	0.9988	0.803

## Data Availability

The data used to support the findings of this study are available from the corresponding author upon request.
